# Editorial: Women in language and computation 2022

**DOI:** 10.3389/frai.2023.1299100

**Published:** 2023-10-17

**Authors:** Róisín Loughran, Huma Shah, Johanna Monti, Maayan Zhitomirsky-Geffet

**Affiliations:** ^1^Regulated Software Research Centre, Dundalk Institute of Technology, Dundalk, Ireland; ^2^EEC School of Computing, Mathematics and Data Sciences, Coventry University, Coventry, United Kingdom; ^3^Department of Literary, Linguistic and Comparative Studies, University of Naples “L'Orientale”, Naples, Italy; ^4^Department of Information Science, Bar-Ilan University, Ramat Gan, Israel

**Keywords:** linguistics, language, women in STEM, computer science, Artificial Intelligence

Women make up < 30% of researchers worldwide. Such under-representation of women are generally most prevalent in technical disciplines such as AI and computer science. Hence, we were delighted to be part of the team to curate this collection of articles in a special edition of Frontiers in Artificial Intelligence to promote female-led, cutting-edge work in the area of Women in Language and Computation.

The past year has witnessed a boom in language and computation with the increasingly controversial public results from Large Language Models (LLMs) such as Google's BARD and OpenAI'sChatGPT now implemented in Microsoft's Bing browser. While LLMs have been well-researched within the academic community for a number of years, there is no denying that high profile, easy to use and freely available tools such as these have pushed this technology into a new level of accessibility and acceptance by the general public. Nonetheless issues of copyright/intellectual property and personal data collection and online privacy abound. While chatbots/conversational AI have been used in e-commerce for more than a decade as virtual customer service assistants, LLMs have now become part of many people's daily lives, assisting them with writing emails, help with school homework and assistance with coding. While this technology poses many advantages, there are some key risks in regards to ethical use of these new tools. Potential threats in regards to plagiarism and detrimental biases have been raised by concerned researchers. In the technical world, which already shows biases against women and other minorities, how can we ensure such biases are not carried into, or even exacerbated, by these new technologies as they now replace traditional search?

Even without these AI technologies, the internet and in particular social media have created a colossal amount of written text that is not evenly distributed across the world. We are therefore very excited to gather articles from women in this field who are addressing a variety of aspects and challenges in language and computation including sentiment analysis, stimuli and pronoun resolution, the space of word meaning, underlying communication issues in LLMs and the problem of hate speech.

Viola from Luxembourg Centre for Contemporary and Digital History looks at a functional grammar in the Italian language to test sentiment analysis in her paper “*On the use of sentiment analysis for linguistics research. Observations on sentiment polarity and the use of the progressive in Italian*.” The article offers a conceptual and methodological contribution to linguistics in exploring the potential benefits and limitations of using a quantitative method such as sentiment analysis for linguistic research.

Repp and Schumacher studied EEG data to investigate the real time resolution of language processing in the brain in their paper “*What naturalistic stimuli tell us about pronoun resolution in real-time processing*.” Their study used naturalistic stimuli considering the unexpectedness of referential expression and the consequences of attentional reorientation.

Piñango examines the communicative illusion we share when considering the meaning of words in “*Solving the elusiveness of word meanings: two arguments for a continuous meaning space for language.”* In this paper she looks at two challenges in evaluating an algebraic continuous system for word meaning.

While LLMs have displayed impressive results in recent years in providing satisfactory answers to inputs or “prompts” there has been little focus on the importance of the underlying communication between agent and person. The aspect of adapting communication and particularly to how a speaker adapts to an interlocutor with different background knowledge is presented in Greco et al.'s paper “*She adapts to her student: an expert pragmatic speaker tailoring her referring expressions to the layman listener.”* This study is particularly focused on how a speaker can learn from a listener's mistakes to adapt their background knowledge.

Hate speech and online abuse is one of the most problematic aspects of social media and the anonymity that comes from interacting with people online. Hilte et al. present their paper “*Who are the haters? A corpus-based demographic analysis of authors of hate speech”* which examines the levels of hate speech in four European languages in relation to two groups at high-risk of hate speech—migrants and the LGTB+ community.

We recognize the great work that is being produced by women in this field and we are happy that Frontiers for AI have created this special edition, but we feel that more should be done. There is still a large disparity in representation and opportunities for women in STEM subjects, especially in computational sciences and it is only when those in positions of influence, such as editors, funding and recruiters, make a conscious effort to address this Research Topic, that equality will be achieved.

## Author contributions

RL: Writing—original draft. HS: Writing—original draft. JM: Writing—review and editing. MZ-G: Writing—review and editing.

